# Short-term clinical and radiographic outcomes of total hip arthroplasty with PMPC-grafted highly cross-linked polyethylene liners against 32-mm femoral heads

**DOI:** 10.1007/s10047-020-01246-0

**Published:** 2021-01-15

**Authors:** Kanto Mouri, Tatsuro Karita

**Affiliations:** Orthopedics Department, Tokyo Metropolitan Tama Medical Center, 2-8-29 Musashidai, Tatsuro KaritaFuchu, Tokyo 183-8524 Japan

**Keywords:** Arthroplasty, Hip prosthesis, Joint replacement, Polyethylene, Wear resistance

## Abstract

We evaluated the clinical and radiographic outcomes of femoral head penetration and total hip arthroplasties with untreated and poly (2-methacryloyloxyethyl phosphorylcholine) (PMPC)-grafted highly cross-linked polyethylene (HXLPE) acetabular liners against 26-, 28-, and 32-mm cobalt–chromium alloy femoral heads 3 years after the index surgery. Three combinations of the articulating surfaces were evaluated in the present study: untreated or PMPC-grafted HXLPE liner against 26- or 28-mm femoral heads (*n* = 16, 24) [control (26 or 28 mm) and PMPC (26 or 28 mm)] and PMPC-grafted HXLPE liner against 32-mm femoral heads (*n* = 64) [PMPC (32 mm)]. The clinical outcomes improved at 3 years postoperatively for the groups. No periprosthetic osteolysis or acetabular component migration was detected, and no revision surgery was performed among the groups. The steady-state wear rate of the PMPC (26 or 28 mm) group (0.021 mm/year) was lower than that of the control (26 or 28 mm) group (− 0.015 mm/year); the steady-state wear was under the clinical threshold. In contrast, the steady-state wear rate of the PMPC (32 mm) group (-0.006 mm/year) showed no significant difference when compared to that of the PMPC (26 or 28 mm) group (*p* < 0.01). The results obtained in the present study clearly demonstrate that PMPC-grafting onto an HXLPE surface improved the wear resistance of acetabular liners, even when coupled with larger femoral heads. Although further follow-up evaluations are required, PMPC-grafted HXLPE acetabular liners may be a promising approach to extend the longevity of artificial joints.

## Introduction

Total hip arthroplasty (THA) is the most frequently performed surgery worldwide, and its clinical results are almost always favorable. However, one of the major reasons for revision surgery after THA is aseptic loosening, which occurs secondarily to periprosthetic osteolysis due to particles that wear, mainly from the polyethylene (PE) acetabular liners [1]. Reductions in worn particle production and bone resorption are the most critical solutions to this problem. To improve wear resistance, the first-generation highly cross-linked PE (HXLPE) acetabular liner was introduced in the late 1990s and quickly became the dominant alternative orthopedic procedure. Positive clinical findings due to wear resistance of the first-generation HXLPE [2] were reported from medium-term follow-ups [3].

Although the first-generation HXLPE decreased wear, Lachiewic et al. reported radiological results of HXLPE at a mean follow-up of 11 years and osteolytic lesions were noted in 12 hips (14%) [4]. There is thus the clinical need to improve the outcomes and delay revision THA surgeries to 20–30 years. This has led to the development of an additional second-generation HXLPE to reduce wear using a method other than irradiation [5]. Understanding the tribology of innate synovial joints and the role of lubrication is important for improving wear resistance in artificial joints. While the bearing surfaces of artificial joints (e.g., HXLPE acetabular liners and metal/ceramic femoral heads) are hydrophobic, the surface of natural synovial joints (i.e., articular cartilage) is hydrophilic and serves as an effective lubricated boundary owing to its nanometer-scaled phospholipid surface layer [6]. Moro et al. developed a surface treatment technology to coat the HXLPE surface of artificial hip joints with a hydrophilic synthetic phospholipid polymer, poly (2-methacryloyloxyethyl phosphorylcholine) (PMPC) [7]. Surface grafting with PMPC, which is a coat of cartilage-mimicking hydrogel structures that are 100–200 nm in thickness, renders the bearing surface of HXLPE acetabular liners hydrophilic and lubricated without affecting the physical and mechanical properties of the HXLPE substrates [8, 9]. Previous hip simulator studies revealed that grafting reduced wear in HXLPE acetabular liners substantially [10]. The results of multicenter clinical trials of THA with PMPC-HXLPE acetabular liners, which begun in 2007 in Japan, reported that at 1 and 5 years postoperatively, the PMPC-HXLPE acetabular liner was an improved option for THA [11, 12]. The clinical trials reported an improvement in the clinical scores, and no revision surgery due to PMPC-HXLPE acetabular liner degradation was required. The rate of wear of the PMPC-HXLPE acetabular liner was 0.002 mm per year, which was significantly lower than of other untreated HXLPE acetabular liners. Although these findings suggested that PMPC-grafted HXLPE acetabular liners may be a promising approach to improve the longevity of artificial hip joints, the report [12] had several limitations. First, only one femoral head size (26 mm) was used for all the clinical trials. Currently, larger femoral heads are being used more frequently to prevent THA dislocation. Second, the study did not have a control group to compare the results of PMPC-HXLPE acetabular liners with untreated HXLPE acetabular liners to evaluate the efficiency of PMPC-grafting.

The aim of the present study was therefore twofold: first, to determine the relative wear rate resulting from the use of the PMPC-HXLPE acetabular liners compared to that from the use of untreated HXLPE acetabular liners, and second, to determine the difference in the relative wear rate of PMPC-HXLPE resulting from the use of 32-, 26-, or 28-mm-sized femoral heads. The use of the 32-mm femoral head was highlighted against a previous report on femoral head sizes.

## Materials and methods

### Patients and implants

A total of 115 hips in 103 patients received a cementless THA system [KYOCERA Medical Corp., Osaka, (currently KYOCERA Corp., Kyoto), JAPAN]. Two kinds of HXLPE acetabular liners were used: PMPC-HXLPE (PMPC) acetabular liners (*n* = 88; Aquala Q3/Q5LP) and untreated HXLPE acetabular liners (*n* = 16; Excellink Q5LP). The PMPC-HXLPE group consisted of 99 hips in 90 patients, which were surgically treated between Sep 2011 and Nov 2013 at our hospital. Eleven hips in eight patients were lost during the follow-up at 3 years postoperatively. Therefore, 88 hips in 82 patients were analyzed (follow-up rate: 82.8%). The untreated HXLPE acetabular liner [control (26/28 mm)] group consisted of 16 hips in 13 patients, which were operated on between May 2010 and Aug 2011 at our hospital, and 16 hips in 16 patients were analyzed (follow-up rate: 100%). The preoperative demographic and implant data for all the groups are shown in Table 1. In all the groups, all operations were performed using the posterior approach.

**Table 1:** Demographic data of the patients using the liner of PMPC (26/28 mm), PMPC (32 mm), and control (26/28 mm)

Items	PMPC (26/28 mm)	PMPC (32 mm)		Control (26/28 mm)	
			*p* value vs PMPC (26/28 mm)		*p* value vs PMPC (26/28 mm)
Sex					
Male	4	8	0.729	1	0.631
Female	20	56		15	
Side					
Right	15	35	0.510	6	0.121
Left	9	29		10	
Preoperative diagnosis					
Osteoarthrosis	21	52	0.865	14	0.361
Osteonecrosis	2	8		2	
Rheumatoid	1	3		0	
Trauma	0	1		0	
Age	64.4 ± 14.2 (34–84)	67.6 ± 11.8 (43–89)	0.220	65.1 ± 8.6 (49–80)	0.916
Body height (cm)	151.0 ± 7.2 (140–167)	156.0 ± 8.4 (138–176)	0.028	153.0 ± 4.7 (148–168)	0.250
Body weight (kg)	52.5 ± 2.4 (38.2–77.5)	57.6 ± 9.7 (39.0–83.0)	0.030	52.8 ± 7.0 (43.0–65.2)	0.993
Body mass index	22.9 ± 2.4 (18.8–27.2)	23.7 ± 3.4 (16.9–27.9)	0.287	22.5 ± 3.3 (18.1–27.9)	0.528

Cobalt–chromium (Co–Cr) alloy femoral heads (K-MAX HH-02; 26-, 28-, and 32-mm head diameters) were used for all the patients. The size distribution of the femoral head is shown in Table 2a. The size distribution of the acetabular shell components is shown in Table 2b. No 32 mm Co–Cr alloy femoral heads were used in the control (26/28 mm) group. Therefore, the PMPC group was subdivided into two groups, the PMPC (26/28 mm) and PMPC (32 mm) groups, to evaluate the effect of PMPC-grafting [PMPC (26/28 mm) vs the control (26/28 mm)] and femoral head diameter [PMPC (26/28 mm) vs PMPC (32 mm)].

**Table 2:** Femoral head diameter and size of acetabular shell component using the liner of PMPC (26/28 mm), PMPC (32 mm), and control (26/28 mm)

Items	PMPC (26/28 mm)	PMPC (32 mm)	Control (26/28 mm)
Femoral head diameter (mm)			
26	1	0	1
28	23	0	15
32	0	64	0
Size of acetabular shell component (mm)			
44	1	0	0
46	5	0	2
48	17	0	6
50	1	34	5
52	0	24	2
54	0	4	0
56	0	1	1
58	0	1	0

An alkali- and heat-treated, porous-coated, titanium alloy collarless femoral stem/acetabular shell component system (AHFIX Q system) was used in the present study. The AHFIX Q3 is a standard-profile acetabular component with no fins. In contrast, the AHFIX Q5LP is a low-profile acetabular component with four fins.

The untreated HXLPE and PMPC-HXLPE acetabular liners used in the present study were designed for the AHFIX Q3 and Q5LP acetabular shell components, respectively. All the liners were highly cross-linked by gamma irradiation at a dose of 50 kGy, heat annealed, and sterilized with 25 kGy of gamma irradiation in nitrogen gas. A difference between the untreated and PMPC-HXLPE acetabular liners was the PMPC-grafting process before the sterilization [8]. Although the small acetabulum used for the Asian population limits the acetabular component size and liner thickness, acetabular liners with a thickness of at least 6.5 mm were used in the present study [13].

All subjects enrolled in this research have given their informed consent, which has been approved by my institutional committee on human research, and this protocol has been found acceptable by them.

### Clinical evaluation

For clinical evaluation, data on demographics, clinical performance, complications, and survival rate from medical records were retrospectively reviewed. Clinical performance was evaluated before surgery and at 3 years postoperatively. The hip joint function chart of the Japanese Orthopaedic Association (JOA score) was used for the evaluation. The JOA score consists of the following four categories: pain (40 points), range of motion (20 points), gait (20 points), and activities of daily living (ADL, 20 points). The sum of the points in these four categories was used to estimate hip function, with a total score of 100 points indicating normal functioning. Fujisawa et al. reported an excellent correlation between the JOA and Harris hip scores (HHS) (coefficient of correlation: 0.843). Therefore, the HHS equivalent was calculated using the following regression formula: HHS = JOA score × 0.979 + 4.363 [14].

### Radiographic evaluation

Anteroposterior non-weight-bearing pelvic and femoral digital radiographs were obtained preoperatively and at 2 weeks, 6 months, and 1 and 3 years postoperatively. The distance between the X-ray tube and the imaging plate was set at 100 cm, and the center of the X-ray beam was directed at the cranial end of the pubic symphysis. To assess the orientation of the acetabular component, the inclination and anteversion angle were measured. The acetabular component inclination angle was defined as the angle between a horizontal line connecting the ischial spines and a line tangential to the opening of the component [15]. The acetabular component anteversion angle was measured using the trigonometric method previously described by Liaw et al. [16].

### Wear rate

An independent engineering researcher measured the position of the femoral head in digitized radiographic images obtained at 2 weeks, 6 months, and 1 and 3 years postoperatively using a computerized algorithm provided by PolyWare software (Draftware Inc., Vevay, IN) featuring a digital, edge-detection algorithm to fit circles and ellipses to the component [17]. The measurements were taken three times and the mean values were taken. Linear wear of the acetabular liners was determined radiographically by the computerized algorithm, which, in addition, calculated the time course of femoral head penetration by tracking head-center movement relative to the acetabular liner center presented by frontal view plain radiographs. This method relies on computer-assisted technology to create a three-dimensional solid model of the acetabular component and femoral head based on a back-projection of the radiographs, femoral head size, and the observer’s knowledge of the design of the acetabular component (software contains a CAD library of various prosthetic brands) [18]. Several authors reported a biphasic pattern in the progression of femoral head penetration into the acetabular liner [2, 19]. In the first phase, the femoral head rapidly moves into the liner by a phenomenon known as “bedding-in,” which is largely attributed to permanent plastic deformation and settling of the liner in the metal shell [19]. Following previous study [12], “bedding-in,” was defined as 1 year operatively. In the second phase, the femoral head slowly moves into the liner; this movement is largely attributed to true wear (material loss in the form of particles) and is considered to define the “steady-state wear rate.” The present report uses these terms to describe the measurement results.

### Statistical analysis

The Student’s *t* test was used to compare the demographic data for each group, and cross-tabulation analysis was used to compare the preoperative diagnosis criteria. The demographic data of each of the three groups were also subjected to cross-tabulation analysis. The JOA score and HHS equivalent before surgery and at postoperative year 3 for each group, as well as the orientation, bedding-in, and steady-state wear rate of the acetabular component, were analyzed using Student’s *t* test. The demographic data and acetabular shell orientation for each group were further divided into two subgroups for comparison, and the correlation of liner thickness, cup orientation, and bedding-in with the steady-state wear rate was calculated. All statistical analyses were performed using add-in software (Bellcurve) on Microsoft Excel. The threshold for significance was *p* < 0.05.

## Results

### Clinical evaluation

For the PMPC (26/28 mm) and PMPC (32 mm) groups, the JOA score was not recorded for two hips of two patients and six hips of six patients, respectively, and was, therefore, assessed for 22 hips in 22 patients and 58 hips in 55 patients, respectively. For all three groups, the mean JOA score and the scores for all four parameters (pain, range of motion, gait, and ADL) significantly increased at 3 years postoperatively (*p* < 0.01; Table 3). Furthermore, the mean HHS equivalent calculated using the Fujisawa’s regression formula significantly improved at 3 years postoperatively in all three groups (*p* < 0.01, Table 3) [14].

**Table 3:** JOA score and HHS equivalent before surgery and 3 years after THA using the liner of PMPC (26/28 mm), PMPC (32 mm), and control (26/28 mm)

Scores	PMPC (26/28 mm)			PMPC (32 mm)					Control (26/28 mm)				
	Before surgery (*1)	3 years after THA (*2)		Before surgery (*3)		3 years after THA			Before surgery (*4)		3 years after THA		
			*p* value vs *1		*p* value vs *1		*p* value vs *3	*p* value vs *2		*p* value vs *1		*p* value vs *4	*p* value vs *2
Total	36.9 ± 12.6	92.4 ± 7.5	< 0.01	37.3 ± 12.5	0.747	91.0 ± 7.0	< 0.01	0.465	37.7 ± 10.1	0.739	93.9 ± 4.7	< 0.01	0.430
Pain	8.4 ± 9.2	38.9 ± 2.1	< 0.01	6.9 ± 8.0	0.634	38.6 ± 2.3	< 0.01	0.513	5.0 ± 6.1	0.249	38.8 ± 2.2	< 0.01	0.600
ROM	12.2 ± 3.3	17.4 ± 2.6	< 0.01	13.0 ± 3.2	0.296	17.3 ± 2.1	< 0.01	0.920	13.3 ± 3.3	0.368	17.4 ± 1.7	< 0.01	0.822
Gait	5.9 ± 4.5	18.2 ± 3.4	< 0.01	6.3 ± 3.6	0.525	17.6 ± 3.3	< 0.01	0.533	7.5 ± 3.1	0.163	18.6 ± 1.9	< 0.01	0.479
ADL	10.4 ± 3.1	18.0 ± 3.3	< 0.01	11.0 ± 3.2	0.513	17.5 ± 2.4	< 0.01	0.571	11.9 ± 2.5	0.130	19.1 ± 1.0	< 0.01	0.160
HHS equivalent	40.5 ± 12.3	94.8 ± 7.3	< 0.01	40.8 ± 12.2	0.747	93.5 ± 6.8	< 0.01	0.465	41.2 ± 14.3	0.739	96.3 ± 9.0	< 0.01	0.430

Two dislocations (cases 36 and 74) in the PMPC (32 mm) group and one dislocation in the control (26/28 mm) group (case 14) occurred within 3 months postoperatively (1, 3, and 2 months, respectively). All dislocations were treated by closed reduction. One periprosthetic fracture of the great trochanter (case 37) occurred in the PMPC (32 mm) group at 18 months postoperatively and was successfully treated with conservative therapy. One superficial surgical site infection (case 3) occurred in the control (26/28 mm) group at 2 weeks postoperatively, and was treated surgically, and the implant was successfully preserved. No adverse events attributable to the implanted PMPC-HXLPE acetabular liner were observed. No revision surgery was required for any of the groups.

### Radiographic evaluation

The mean inclination and anteversion angles of the acetabular component are 42.0 ± 7.1 and 18.5 ± 7.8 in the PMPC (26/28 mm) group, 43.4 ± 6.4 and 19.0 ± 7.9 in the PMPC (32 mm) group, and 44.1 ± 8.6 and 17.8 ± 6.8 in the control (26/28 mm) group. In all three cases of dislocation, the acetabular components were implanted outside the Lewinnek safe zone [20].

### Wear rate

The mean bedding-in (mm) of the PMPC (26/28 mm), PMPC (32 mm), and control (26/28 mm) group was 0.298 ± 0.130, 0.315 ± 0.121, and 0.306 ± 0.121 mm, respectively. There was no significant difference between the PMPC (26/28 mm) and PMPC (32 mm) groups (*p* = 0.445) or the PMPC (26/28 mm) and control (26/28 mm) groups (*p* = 0.669). The three groups did not show any association with patient, surgical, or implant factors (Tables 4, 5).

**Table 4:** Associations between the bedding-in and demographic data, and surgical factors

	PMPC (26/28 mm)					PMPC (32 mm)					Control (26/28 mm)				
	Group	Bedding-in	Group	Bedding-in		Group	Bedding-in	Group	Bedding-in		Group	Bedding-in	Group	Bedding-in	
					*p* value					*p* value					*p* value
Age	≤ 68 (*n* = 12)	0.329 ± 0.154	≥ 69 (*n* = 12)	0.267 ± 0.081	0.242	≤ 68 (*n* = 32)	0.306 ± 0.107	≥ 69 (*n* = 32)	0.323 ± 0.130	0.992	≤ 68 (*n* = 10)	0.318 ± 0.126	≥ 69 (*n* = 6)	0.287 ± 0.096	0.539
Body mass index	≤ 23.0 (*n* = 13)	0.280 ± 0.137	≥ 23.1 (*n* = 11)	0.319 ± 0.109	0.700	≤ 23.0 (*n* = 31)	0.309 ± 0.101	≥ 23.1 (*n* = 33)	0.320 ± 0.134	0.716	≤ 23.0 (*n* = 11)	0.318 ± 0.125	≥ 23.1 (*n* = 5)	0.280 ± 0.090	0.466
Body height (cm)	≤ 152 (*n* = 15)	0.282 ± 0.077	≥ 153 (*n* = 9)	0.324 ± 0.178	0.306	≤ 152 (*n* = 25)	0.333 ± 0.125	≥ 153 (*n* = 39)	0.303 ± 0.114	0.370	≤ 153 (*n* = 7)	0.254 ± 0.083	≥ 154 (*n* = 9)	0.347 ± 0.122	0.098
Body weight (kg)	≤ 53.9 (*n* = 16)	0.301 ± 0.129	≥ 54.0 (*n* = 8)	0.292 ± 0.123	0.642	≤ 53.9 (*n* = 22)	0.344 ± 0.129	≥ 54.0 (*n* = 42)	0.299 ± 0.111	0.269	≤ 54.5 (*n* = 10)	0.310 ± 0.129	≥ 54.9 (*n* = 6)	0.300 ± 0.094	0.659
Diagnosis	OA (*n* = 21)	0.294 ± 0.121	Other (*n* = 3)	0.330 ± 0.158	0.548	OA (*n* = 52)	0.312 ± 0.128	Other (*n* = 12)	0.326 ± 0.074	0.667	OA (*n* = 14)	0.311 ± 0.110	Other (*n* = 2)	0.274 ± 0.150	–
Cup inclination(°)	30–44 (*n* = 17)	0.301 ± 0.302	≥ 45 (*n* = 7)	0.325 ± 0.117	0.696	30–44 (*n* = 39)	0.315 ± 0.123	≥ 45 (*n* = 25)	0.314 ± 0.114	0.655	30–44 (*n* = 7)	0.320 ± 0.135	≥ 45 (*n* = 9)	0.296 ± 0.099	0.659
Cup anteversion(°)	5–25 (*n* = 18)	0.308 ± 0.135	< 5, > 25 (*n* = 6)	0.267 ± 0.093	0.344	5–25 (*n* = 39)	0.318 ± 0.124	< 5, > 25 (*n* = 15)	0.303 ± 0.103	0.835	5–25 (*n* = 15)	0.308 ± 0.120	< 5, > 25 (*n* = 1)	0.275	–

**Table 5:** Coefficient of correlation between bedding-in, steady wear rate, and surgical factors

	PMPC (26/28 mm)		PMPC (32 mm)		Control (26/28 mm)	
	Bedding-in	Steady wear rate	Bedding-in	Steady wear rate	Bedding-in	Steady wear rate
Liner thickness (mm)	0.02	− 0.13	0.02	0.09	0.29	− 0.05
Cup inclination (°)	− 0.09	− 0.18	− 0.01	− 0.08	0.40	− 0.23
Cup anteversion (°)	0.05	0.07	− 0.14	− 0.11	− 0.54	− 0.05

For the PMPC (26/28 mm), the PMPC (32 mm), and the control (26/28 mm) groups, 66.7, 45.3, and 32.5% showed negative wear values, respectively. The mean steady-state wear rate of the PMPC (26/28 mm) group (− 0.015 ± 0.056 mm/year range: − 0.0135–0.081 mm) improved compared to that of the control (26/28 mm) group (0.021 ± 0.069 mm/year range: − 0.131–0.142 mm), although/the difference was not statistically significant (*p* = 0.064, effect size: *d* = 0.57, 1−*β* = 0.41). In contrast, there was no significant difference between the mean steady-state wear rate of the PMPC (26/28 mm) and PMPC (32 mm) groups (− 0.006 ± 0.058 mm/year range: − 0.156 mm–0.144 mm; *p* = 0.707, effect size: *d* = 0.158, 1−*β* = 0.100). None of the groups showed any association between the mean steady-state wear rate and patient, surgical, or implant factors (Tables 5, 6). Previously, the measurements were taken three times by one person, and intra-correction coefficients of the observer was 0.390.

**Table 6:** Associations between the steady-state wear rate and demographic data, and surgical factors

	PMPC (26/28 mm)					PMPC (32 mm)					Control (26/28 mm)				
	Group	Steady wear rate	Group	Steady wear rate		Group	Steady wear rate	Group	Steady wear rate		Group	Steady wear rate	Group	Steady wear rate	
					*p* value					*p* value					*p* value
Age	≤ 68 (*n* = 12)	− 0.013 ± 0.044	≥ 69 (*n* = 12)	− 0.018 ± 0.064	0.805	≤ 68 (*n* = 32)	− 0.012 ± 0.061	≥ 69 (*n* = 32)	− 0.001 ± 0.054	0.330	≤ 68 (*n* = 10)	0.018 ± 0.069	≥ 69 (*n* = 6)	0.027 ± 0.062	0.823
Body mass index	≤ 23.0 (*n* = 13)	− 0.012 ± 0.056	≥ 23.1 (*n* = 11)	− 0.019 ± 0.054	0.884	≤ 23.0 (*n* = 31)	0.002 ± 0.063	≥ 23.1 (*n* = 33)	− 0.014 ± 0.052	0.145	≤ 23.0 (*n* = 11)	0.033 ± 0.074	≥ 23.1 (*n* = 5)	− 0.004 ± 0.035	0.191
Body height (cm)	≤ 152 (*n* = 15)	− 0.026 ± 0.057	≥ 153 (*n* = 9)	0.002 ± 0.047	0.362	≤ 152 (*n* = 25)	− 0.013 ± 0.053	≥ 153 (*n* = 39)	− 0.002 ± 0.060	0.640	≤ 153 (*n* = 7)	0.037 ± 0.068	≥ 154 (*n* = 9)	0.009 ± 0.063	0.245
Body weight (kg)	≤ 53.9 (*n* = 16)	− 0.023 ± 0.054	≥ 54.0 (*n* = 8)	0.000 ± 0.053	0.257	≤ 53.9 (*n* = 22)	− 0.007 ± 0.053	≥ 54.0 (*n* = 42)	− 0.006 ± 0.054	0.908	≤ 54.5 (*n* = 10)	0.037 ± 0.077	≥ 54.9 (*n* = 6)	− 0.004 ± 0.032	0.458
Diagnosis	OA (*n* = 21)	− 0.014 ± 0.057	Other (*n* = 3)	− 0.027 ± 0.036	0.283	OA (*n* = 52)	− 0.007 ± 0.059	Other (*n* = 12)	− 0.006 ± 0.053	0.976	OA (*n* = 14)	0.024 ± 0.049	Other (*n* = 2)	0.006 ± 0.137	–
Cup inclination(°)	30–44 (*n* = 17)	− 0.012 ± 0.056	≥ 45 (*n* = 7)	− 0.038 ± 0.044	0.320	30–44 (*n* = 39)	− 0.003 ± 0.055	≥ 45 (*n* = 25)	− 0.012 ± 0.062	0.167	30–44 (*n* = 7)	0.029 ± 0.039	≥ 45 (*n* = 9)	0.015 ± 0.081	0.859
Cup anteversion(°)	5–25 (*n* = 18)	− 0.013 ± 0.054	< 5, > 25 (*n* = 6)	− 0.024 ± 0.058	0.956	5–25 (*n* = 39)	− 0.005 ± 0.060	< 5, > 25 (*n* = 15)	− 0.012 ± 0.050	0.559	5–25 (*n* = 15)	0.022 ± 0.069	< 5, > 25 (*n* = 1)	0.008	–

## Discussion

The present study evaluated the clinical and radiographic outcomes, 3 years postoperatively, of cementless primary THA using PMPC-HXLPE acetabular liners. In the PMPC-HXLPE acetabular liner (26/28 mm) group, the mean HHS equivalent improved during the 3 postoperative years, showing no significant difference when compared to the control (26/28 mm) group. In addition, the clinical outcomes of the present study were similar to those of other reports of THA using 26- or 28-mm Co–Cr alloy femoral heads with HXLPE or PMPC-HXLPE acetabular liners [12, 19]. In the PMPC (32 mm) group, the mean HHS equivalent improved during the postoperative 3 years, showing no significant difference when compared with the PMPC (26/28 mm) group. As previously noted, for untreated HXLPE liners [21], large femoral heads did not affect the improvement in the clinical scores for PMPC-HXLPE liners.

The use of larger femoral heads has recently become common as a result of improve stability in THA, and larger femoral heads reduce the dislocation rate [22]. However, in the present study, dislocation occurred in two cases in the PMPC (32 mm) group, while no dislocation occurred in the PMPC (26/28 mm) group. In both cases, the acetabular components were implanted outside the Lewinnek safe zone [20]. Therefore, the position of the acetabular component might have caused the dislocation. In addition, 64 individuals may have been an insufficient sample number to exclude the possibility of rare complications.

The early bedding-in of the femoral head resulting from the depression of PE and other factors can confound early wear measurements. Digas et al. [23] reported a similar magnitude of bedding-in which was complete at postoperative year 1; it was thus considered reasonable to define the period required for the bedding-in phase as 1 postoperative year. Femoral head penetration occurring after this time was thus representing true wear. In the present study, there was no significant difference in bedding-in between the PMPC (26/28 mm) and control (26/28 mm) groups (Table 4). As the demographic data of the two groups did not differ significantly (Table 1, 2), this result suggests that the PMPC-grafting had no effect on the physical or mechanical properties of the HXLPE substrate, which is congruent with the findings of a previous study [8].

There was a difference in the steady-state wear between the PMPC (26/28 mm) and control (26/28 mm) groups. The demographic data of the two groups did not differ significantly (Table 1); this suggests that PMPC-grafting improved wear resistance of the HXLPE liner. In previous reports using radiographic measurement techniques based on clinical X-rays, a negative wear rate was a common issue, especially in short-term studies of recent HXLPE liners [2, 5]. This negative wear rate was considered to be as a result of the accuracy limitations associated with radiographic measurement techniques [2, 5, 18]. Engh et al. reported a negative wear rate in 32% of the patients [2]; negative wear rates were included in analysis under the assumption that the measurement error was randomly distributed, and the measurement error in the negative penetration direction and in the positive direction was equal. Therefore, the present study also included the negative wear rate in its analysis.

The use of larger femoral heads is a trade-off between increased stability and decreased THA survivorship [24]. These femoral heads lead to a higher wear rate than that of smaller femoral heads. Moreover, a larger femoral head requires a thinner PE liner, which may create high contact stress and possibly accelerate wear [25]. However, in the present study, there was no significant difference in the mean steady-state wear rate in the PMPC (26/28 mm) and PMPC (32 mm) groups. The demographic data of the two groups did not differ significantly (Table 1), which suggests that PMPC-grafting improved the wear resistance of the HXLPE liner, including when coupled with a larger femoral head.

There are several limitations to our study. First, this study was not a randomized-controlled trial. A randomized-controlled trial comparing PMPC-HXLPE and untreated HXLPE liners may be a method for evaluating the efficiency of PMPC-treated bearing surfaces. However, in this study, the demographic data of the patients in the PMPC-HXLPE and untreated HXLPE liner groups did not differ significantly. This drawback might be offset by the evaluations in this study. Second, 88 individuals and a 3-year follow-up may have been insufficient to exclude the possibility of rare adverse reactions related to the new bearing particularly when evaluating very limited wear. The comparisons of wear rate between each group have low statistic power. Some of the clinical scores were not included or presented. Further studies, including long-term follow-ups of the present cohort, are still required to determine the clinical efficacy of the PMPC-grafted bearing surfaces. Third, radiostereometric analysis, which is reportedly the most accurate tool for assessing in vivo wear in acetabular liners, was not performed, because it requires the placement of marker balls. Many potential candidates believed that these marker balls provided no benefit. Therefore, the PolyWare technique was used instead, which reportedly tends to show a higher mean PE wear rate than radiostereometric analysis [26].

## Conclusion

Although the clinical outcomes of the PMPC-HXLPE and untreated HXLPE acetabular liners were similar, the PMPC-HXLPE liners demonstrated markedly lower wear than the untreated HXLPE acetabular liners at the 3-year follow-up, and the steady-state wear remained under the clinical threshold. Furthermore, PMPC-grafting improved wear resistance of the HXLPE liners, even when coupled with larger femoral heads. Further follow-up evaluations are required to determine whether PMPC-HXLPE acetabular liners improve long-term clinical and radiographic outcomes.
